# A Moderated Mediation Analysis of Timely EMS Activation and Bystander CPR in the Association Between Regional Deprivation and Outcomes Following Out-of-Hospital Cardiac Arrest

**DOI:** 10.3390/healthcare14030408

**Published:** 2026-02-05

**Authors:** So Yeon Kong, Seungmin Jeong

**Affiliations:** 1 Laerdal Medical, 4002 Stavanger, Norway; joyce.kong@laerdal.com; 2 Department of Social and Preventive Medicine, Hallym University College of Medicine, Chuncheon 24252, Republic of Korea; 3 Institute of Social Medicine, Hallym University, Chuncheon 24252, Republic of Korea; 4 Gangwon Center for Infectious Diseases, Chuncheon 24280, Republic of Korea

**Keywords:** out-of-hospital cardiac arrest, regional deprivation, emergency medical services, awareness time interval, causal mediation analysis, bystander CPR

## Abstract

**Background/Objectives**: Out-of-hospital cardiac arrest (OHCA) outcomes remain poor and vary widely across communities with socioeconomic deprivation. This study examines whether delays in emergency medical services (EMS) activation, the earliest link in the Chain of Survival, mediate the association between regional deprivation and OHCA outcomes, and whether this effect is modified by bystander cardiopulmonary resuscitation (CPR) status. **Methods**: We analyzed adult patients (aged 18–80 years) with witnessed, EMS-treated OHCA of presumed cardiac etiology from the Korean nationwide OHCA registry (2015–2022). Regional deprivation was defined by the Regional Deprivation Index and dichotomized into deprived (top 20%) vs. non-deprived areas. Timely EMS activation, defined as collapse to EMS activation, was measured as an awareness time interval (ATI) < 5 min. Outcomes were good neurological recovery (CPC 1–2) and survival to discharge. Causal mediation analysis within the counterfactual framework quantified the proportion of the association mediated by timely EMS activation, with stratification by bystander CPR status. **Results**: Among 43,032 patients, 6.1% resided in deprived areas. Deprived areas had lower bystander CPR (22.6% vs. 36.3%) and timely EMS activation (67.8% vs. 75.6%) (*p* < 0.05 for all). Regional deprivation was associated with poorer outcomes (good neurological prognosis: aOR 0.46, 95% CI 0.39–0.55; survival: aOR 0.65, 95% CI 0.57–0.73). Mediation analysis showed that ATI < 5 min accounted for 3.7% of the total deprivation effect on good neurological outcome and 7.9% on survival, with stronger mediation among patients receiving bystander CPR (7.9% and 14.7%, respectively). **Conclusions**: Regional deprivation is significantly associated with poorer OHCA outcomes, partly mediated by delays in EMS activation, particularly among patients who received bystander CPR. Interventions to enhance early recognition, rapid EMS activation, and bystander CPR in deprived communities are critical to improving survival equity after OHCA.

## 1. Introduction

Out-of-hospital cardiac arrest (OHCA) remains a leading cause of mortality worldwide [1,2]. Despite global efforts and significant advances in prevention and treatment over the last several decades, outcomes remain poor, largely due to a lack of timely emergency care for the victims, including early recognition and prompt activation of the emergency medical systems (EMS) [3,4]. Moreover, there is great variation in the survival of OHCA. Much of this variation is attributed to differences across the Chain of Survival [5], a sequence of critical, interconnected actions that maximize the chance of survival after cardiac arrest. The Chain of Survival emphasizes that early recognition and EMS activation is the first and most critical link with the highest life-saving potential [6,7].

For decades, international resuscitation guidelines, such as those from the International Liaison Committee on Resuscitation (ILCOR) and the European Resuscitation Council (ERC), have recommended that lay rescuers call local emergency services after checking responsiveness and abnormal breathing [8,9]. However, the 2025 ERC Guidelines introduced a key update to the Basic Life Support (BLS) algorithm—lay rescuers are now advised to activate EMS immediately after checking responsiveness, without confirming abnormal breathing [10]. This update reflects growing recognition that delays in EMS activation significantly affect outcomes and that early activation should take priority, even before breathing assessment.

Socioeconomic disparities have been increasingly recognized as important determinants of OHCA outcomes, with community-level deprivation associated with lower rates of bystander cardiopulmonary resuscitation (CPR) and poorer survival, highlighting the profound impact of social and community determinants on OHCA outcomes [11,12,13]. One critical factor contributing to these disparities lies in time intervals along the early links of the Chain of Survival. Previous studies suggest that OHCA outcomes are inversely proportional to time from witness to EMS activation, so-called “awareness time” [4,14,15]. Every 1 min delay of call was associated with about a 9% decrease in good neurological recovery and a 7% decrease in survival [16]. While longer awareness time has been linked to worse outcomes, the mediating role of this interval in the relationship between deprivation and clinical outcomes of OHCA remains poorly understood. Additionally, the extent to which bystander CPR modifies this pathway has not been fully elucidated.

This study aims to examine whether awareness time mediates the association between neighborhood deprivation and clinical outcomes of OHCA. We hypothesized that residents in deprived areas experience longer awareness time intervals (ATI) due to potential barriers in symptom recognition or calling for EMS, and that this prolonged ATI serves as a key mediator that accounts for a significant portion of the survival gap between deprived and non-deprived areas. By applying mediation analysis, we seek to quantify the indirect effect of deprivation through awareness time and to better understand how contextual factors contribute to disparities in cardiac arrest survival. Understanding these pathways could inform community-level interventions to enhance early recognition and EMS activation, ultimately improving equity in resuscitation outcomes.

## 2. Materials and Methods

### 2.1. Data Source

This study used data from the Korean nationwide OHCA registry maintained by the Korea Disease Control and Prevention Agency (KDCA) for the period from 2015 to 2022. The registry is a nationwide, population-based OHCA surveillance system. The registry was established in collaboration with the National Fire Agency (NFA) to collect standardized epidemiologic information on OHCA events, monitor survival, and evaluate the performance of the EMS system [17]. All OHCA cases in which patients are transported by the national 119 EMS to a medical institution are captured in this registry, corresponding to approximately 30,000 cases annually [18]. During EMS operations, dispatch and prehospital information are recorded in multiple linked databases, including EMS run sheets, an OHCA in-depth registry, dispatcher-assisted CPR records, and the “pumbulance (fire engine)” registry for multi-unit responses [19]. The registry is structured to align with Utstein-style definitions and links dispatch/prehospital datasets with standardized in-hospital outcome abstraction. Trained medical record reviewers then abstract in-hospital treatment and outcome data from all receiving hospitals using standardized forms, with ongoing quality management through regular audits and feedback [20].

### 2.2. Study Population

From the KDCA OHCA registry, we identified all EMS-treated OHCAs that occurred between 1 January 2015 and 31 December 2022. We included adult patients aged 18–80 years with witnessed OHCA of presumed cardiac etiology who were transported to a medical institution by EMS. We excluded arrests that occurred in hospitals, clinics, ambulances, or nursing homes to focus on community-based events. Among these, we further excluded cases without a bystander witness, cases with missing information on clinical outcomes or ATI, and those with ATI exceeding 30 min, given that such extreme values are likely to reflect recording errors or events outside a plausible window for effective bystander response [4,21].

### 2.3. Measures

#### 2.3.1. Exposure Variable: Regional Deprivation

The main exposure was neighborhood socioeconomic deprivation at the location of the OHCA event, operationalized using the Regional Deprivation Index (RDI). Using 2020 Population and Housing Census data from Statistics Korea, nine area-level indicators reflecting social and material disadvantage were considered, including the proportions of female-headed households, individuals living alone, car-less households, divorced or widowed individuals, low socioeconomic status (SES) households, residents aged >65 years, households with substandard housing conditions, individuals with less than secondary education, and homeownership [22,23,24,25]. For each of the 252 administrative districts, indicators were standardized as z-scores and subjected to principal component analysis with varimax rotation to identify the most salient contributors to deprivation. The final RDI was calculated as the sum of the selected z-score indicators for each district. Following prior work and to facilitate mediation analysis, we dichotomized districts so that those in the highest 20th percentile of the RDI distribution were classified as “deprived areas,” and the remaining 80% were classified as “non-deprived (reference) areas” [26,27].

#### 2.3.2. Outcome Variables: Good Neurological Outcome and Survival to Discharge

The primary outcome was a good neurological outcome, and the secondary outcome was survival to discharge. A good neurological outcome was defined as a favorable cerebral performance at hospital discharge (Cerebral Performance Category [CPC] 1–2), in line with Utstein-style definitions routinely used in the Korean registry. Survival to discharge was defined as being alive at discharge from the emergency department (ED) or after hospital admission, including discharge home, transfer to another facility, or discharge against medical advice. All other cases were categorized as poor neurological outcome and non-survival, respectively, based on the final disposition recorded in the registry.

#### 2.3.3. Mediator: Awareness Time Interval (ATI)

The mediator of interest was the ATI, defined as the time from witnessed collapse to EMS activation (119-emergency call), as recorded in the EMS dispatch system. Delay in the ATI is recognized as a critical determinant of outcomes in OHCA [14]. While direct research linking regional deprivation to ATI remains scarce in the existing literature, it has been established that rural areas experience significant reporting delays compared to urban centers [28]. Furthermore, disadvantaged regions are characterized by prolonged response times. Given that previous studies have shown both response time and time to bystander CPR to be proportional to ATI, it is reasonable to suggest that ATI serves as a plausible mediating variable between regional deprivation and OHCA prognosis [14]. Upon arrival at the scene, EMS providers ascertain the collapse time by directly asking the bystander and record this information. Based on prior literature and current resuscitation guidelines emphasizing rapid activation of EMS, as well as the distribution of ATI in our data, we constructed a binary mediator indicating whether ATI was <5 min (timely call) versus >5 min (delayed call) [4,21]. For all analyses, cases with ATI > 30 min were excluded as described above.

#### 2.3.4. Moderator: Bystander CPR

Bystander CPR was defined as chest compressions performed by laypersons or non-EMS personnel before EMS arrival, as documented in EMS records according to Utstein guidelines. Bystander CPR was treated as a dichotomous variable (yes/no) and was used as an effect modifier. All main analyses were conducted in the overall cohort and then stratified by bystander CPR status to explore whether the mediating role of ATI differed between patients who did and did not receive bystander CPR.

#### 2.3.5. Covariates

Potential confounders were selected a priori based on previous studies of OHCA outcomes and the conceptual framework linking area deprivation, time to call, and clinical outcomes [16,21,22]. Covariates included age, sex, calendar year of OHCA, insurance type (as a proxy for individual socioeconomic position), location of arrest, and the presence of a shockable rhythm before ED arrival. All covariates were measured at the time of the index OHCA event and included in multivariable regression and mediation models.

### 2.4. Statistical Analysis

We described the characteristics of the study’s overall population and by regional deprivation status (deprived vs. non-deprived). Categorical variables were summarized as frequencies and percentages and compared using χ^2^ tests.

To examine the association between regional deprivation and each clinical outcome, we fitted multivariable logistic regression models with deprived area as the main independent variable, separately for good neurological outcome and survival to discharge. For each outcome, we constructed two models: Model 1 adjusted for age group, sex, calendar year, insurance type, location of arrest, and initial ECG rhythm; Model 2 additionally adjusted for the mediator (binary ATI < 5 min), allowing us to observe how much of the deprivation–outcome association was attenuated after accounting for the ATI. To assess effect modification by bystander CPR, we repeated these models stratified by bystander CPR status.

**Figure 1:**
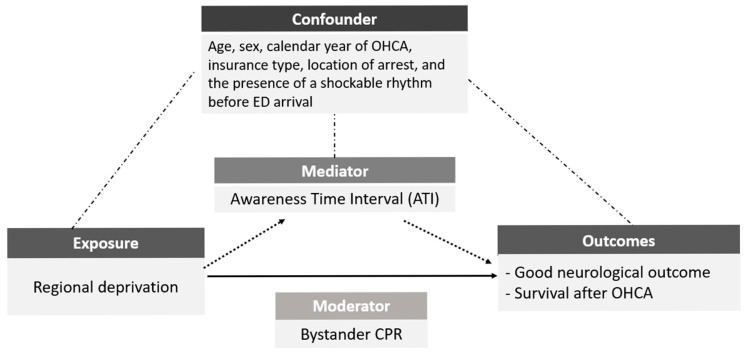
Conceptual analysis model: OHCA = out-of-hospital cardiac arrest; ED = emergency department; ATI = awareness time interval; CPR = cardiopulmonary resuscitation.

We then applied a causal mediation analysis within the counterfactual framework to decompose the total effect of regional deprivation on OHCA outcomes into natural direct and natural indirect effects operating through ATI. For each outcome (good neurological outcome and survival to discharge), we specified a binary exposure (deprived vs. non-deprived area), binary mediator (ATI < 5 vs. ≥5 min), and binary outcome. We used logistic regression models for both the mediator and the outcome, adjusting for the same set of covariates as in Model 1 above. We calculated the proportion mediated as the percentage of the total effect attributable to the indirect pathway via ATI (Figure 1). Conventional mediation analysis typically quantifies the indirect (mediated) effect by comparing regression coefficients from models fit with and without the mediator. However, prior methodological work has shown that such traditional approaches can yield biased estimates of mediation, either inflating or attenuating the true mediated effect when there is confounding between the mediator and outcome, interaction between the exposure and mediator, or mediator–outcome confounding that is itself affected by the exposure [29,30,31]. To overcome these limitations, we employed a causal mediation approach within the counterfactual framework, which offers more robust and flexible estimation of mediation effects in the presence of complex confounding structures and exposure–mediator interactions. To explore whether mediation differed by bystander CPR status, all mediation analyses were conducted in the total cohort and then repeated after stratification by the presence or absence of bystander CPR.

To evaluate the robustness of our findings to alternative operationalizations of early EMS activation, we conducted sensitivity analyses using different ATI cut-points as mediators. Specifically, we redefined the binary mediator as ATI < 3 min, ATI < 4 min, and ATI < 10 min (each vs. longer intervals) and repeated the causal mediation analyses for both outcomes in the overall cohort and within strata of bystander CPR status. The total and direct effects of regional deprivation, as well as the estimated mediation proportions, were compared across these alternative definitions to assess the stability of our conclusions.

All analyses were conducted using SAS software (version 9.4; SAS Institute Inc., Cary, NC, USA), including the CAUSALMED procedure for mediation analysis. Statistical significance was defined as a two-sided *p*-value < 0.05.

### 2.5. Ethics Approval and Consent to Participate

This study was approved by the Institutional Review Board of Hallym University (HIRB-2025-010).

## 3. Results

**Figure 2:**
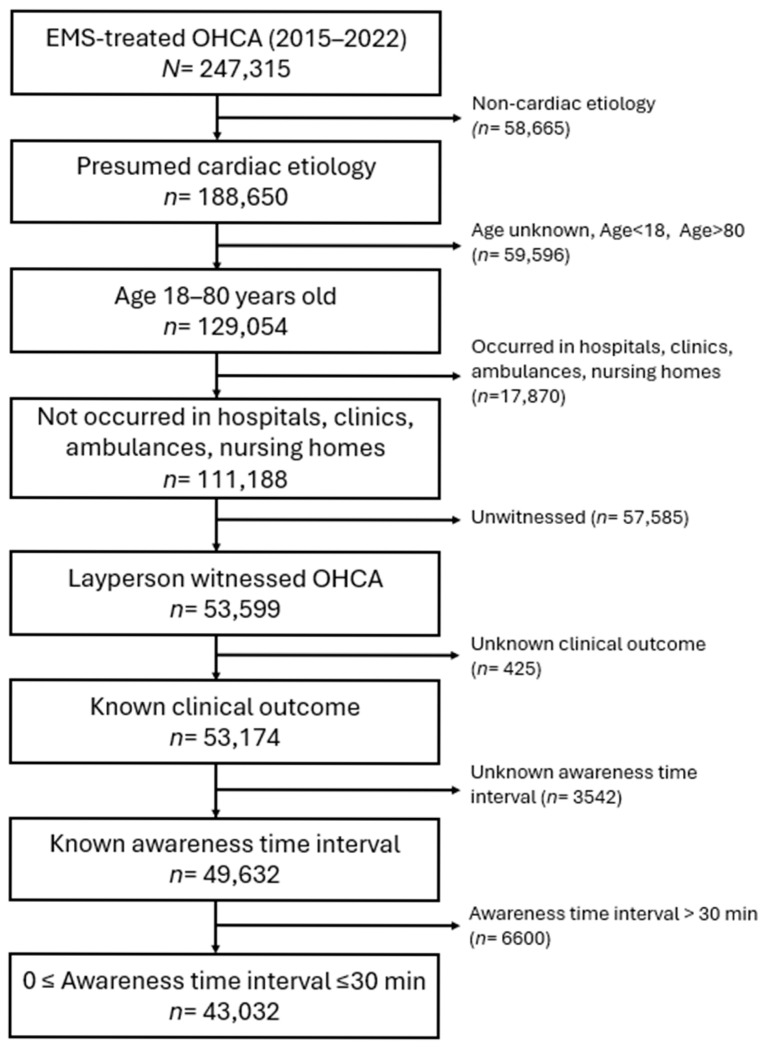
Flow of participants. Abbreviations: EMS = emergency medical services; N = number; OHCA = out-of-hospital cardiac arrest.

Figure 2 shows the study participant flow. Among 247,315 EMS-treated OHCA cases between 2015 and 2022, 188,650 presumed to be of cardiac etiology and were initially included, and the cohort was then restricted to patients aged 18–80 years (*n* = 129,054). We subsequently excluded arrests occurring in hospitals, clinics, ambulances, or nursing homes, yielding 111,188 community-based events. From these, we sequentially excluded cases without a bystander witness, those with missing information on clinical outcomes or awareness-to-call time, and those with an awareness-to-call interval exceeding 30 min. The final analytic sample, therefore, comprised 43,032 adult OHCA patients.

Among a total of 43,032 adult OHCA patients, 2642 (6.1%) were classified as residing in deprived areas. Compared with non-deprived areas, the deprived areas had an older average age, a higher proportion of patients aged 71–80 years (34.3% vs. 42.6%), and slightly higher rates of medical aid and other/unknown insurance categories (*p* < 0.05 for both). OHCAs occurring in public locations were more frequent in non-deprived areas (22.3% vs. 19.2%). The overall rate of bystander CPR was 35.5%, with a significantly lower rate in deprived areas (22.6%) compared to non-deprived areas (36.3%) (*p* < 0.05). The proportion of cases with an awareness time interval (ATI) less than 5 min was also significantly lower in deprived areas (67.8%) than in non-deprived areas (75.6%). Clinical outcomes of good neurological prognosis (6.3% vs. 13.3%) and survival to hospital discharge (14.0% vs. 21.0%) were also statistically worse in deprived areas compared with in non-deprived areas (*p* < 0.05 for both) (Table 1). More detailed information is shown in Supplementary Table S1.

Supplementary Table S2 presents the statistical association between the independent (regional deprivation) and mediating (achieving <5 min of ATI) variables. Multivariable logistic regression analysis demonstrated that deprived areas had significantly lower odds of achieving an ATI < 5 min compared to reference areas. This association was consistent across all subgroups, with an adjusted odds ratio (aOR) of 0.67 (95% CI 0.64–0.76) for the total population, 0.70 (95% CI 0.64–0.78) for cases without bystander CPR, and 0.67 (95% CI 0.56–0.81) for cases with bystander CPR.

In the multivariable logistic regression analyses (Table 2), OHCA patients in a deprived area was significantly associated with poorer clinical outcomes with lower odds of good neurological prognosis (aOR 0.45, 95% CI 0.38–0.54) and survival to discharge (aOR 0.63, 95% CI 0.56–0.71) compared with OHCA patients in non-deprived areas (Model 1). Similar results were observed after additional adjustment for ATI < 5 min in Model 2 (good neurological prognosis, aOR 0.46, 95% CI 0.39–0.55; survival to discharge, aOR 0.65, 95% CI 0.57–0.73).

In stratified analyses by bystander CPR status (Table 2), the association between area deprivation and poor outcomes was more pronounced among those who did not receive bystander CPR. In this group, OHCA patients in deprived areas had significantly lower odds of good neurological outcomes (aOR 0.31; 95% CI, 0.24–0.40) and survival to discharge (aOR 0.59; 95% CI, 0.50–0.69) (Model 2). Among patients who received bystander CPR, the adverse association between area deprivation and outcomes remained significant but was attenuated (good neurological outcome: aOR 0.67, 95% CI, 0.53–0.85; survival to discharge: aOR 0.79, 95% CI, 0.64–0.97).

In the causal mediation analysis (Table 3), OHCA patients in a deprived area were associated with reduced odds of favorable outcomes across all subgroups. For good neurological outcome, the proportion of the total effect mediated by an awareness time interval (ATI) less than 5 min was estimated at 3.65% (95% CI 2.09–5.20) in the total cohort, 2.09% (95% CI 0.99–3.19) among patients without bystander CPR, and 7.92% (95% CI 1.19–14.64) among those with bystander CPR. For survival to discharge, the corresponding mediation proportions were 7.88% (95% CI 4.58–11.18), 6.21% (95% CI 3.26–9.16), and 14.69% (95% CI 0.73–28.64), respectively. 

In sensitivity analyses using alternative ATI thresholds (<3, <4, and <10 min) as mediators (Supplementary Table S3), the total and direct effects of area deprivation on outcomes remained stable, and mediation proportions also remained highly consistent across models, confirming the stability of the findings. The greatest mediation effect was observed when ATI < 10 min was applied, with proportions of 14.2% for good neurological outcome and 23.47% for survival to discharge among patients who received bystander CPR.

## 4. Discussion

In this nationwide registry-based study, OHCA patients from deprived areas were significantly associated with poorer outcomes, both in good neurological recovery and survival to discharge, compared with those from non-deprived areas. Part of this disparity was explained by delays in early emergency recognition and EMS activation, reflected by a longer ATI. An ATI shorter than 5 min mediated a modest yet consistent proportion of the total effect, accounting for approximately 4% of the disparity in good neurological recovery and 8% in survival outcomes in the overall cohort. The mediating effect was more pronounced among patients who received bystander CPR (8% and 15%, respectively).

Our findings align with previous studies demonstrating that lower SES is associated with delayed EMS activation, lower bystander CPR rates, and worse OHCA outcomes across various global settings [32,33,34,35,36,37,38,39]. Time to call EMS and the provision of bystander CPR are among the strongest predictors of survival after OHCA. Studies showed a socioeconomic gradient with these factors, suggesting that individuals with higher SES are benefiting from access to more prompt and effective care by knowledgeable bystanders [34,35].

OHCA is among the most time-sensitive emergencies, with each minute of delay profoundly affecting outcomes. Delays in emergency recognition and EMS activation propagate through subsequent delays in the subsequent links in the Chain of Survival, including initiation of dispatcher-assisted (DA) CPR, EMS response, and hospital arrival. In a study, Zhang et al. reviewed 151 OHCA dispatch records from a metropolitan city EMS in China, one of the earliest EMS centers that has implemented DA-CPR in China, and reported median delays of up to 30 min before contacting EMS, resulting in no survival despite a DA-CPR rate of 73% [40]. The caller delay of 30 min to call for EMS far exceeded the time window for bystander CPR, thus resulting in no survival, showing how extreme caller delay can negate potential benefits of later links of the Chain of Survival. Authors attributed these extreme delays largely to a lack of local and national efforts for public education on recognizing life-threatening emergencies and calling the EMS for help.

The stronger mediation in the bystander CPR subgroup may reflect several distinct but complementary mechanisms. First, the presence of bystander CPR likely indicates earlier recognition of cardiac arrest and greater readiness to respond, which may be accompanied by faster EMS activation [41]. Second, variability in the sequence of early actions (e.g., initiating chest compressions before placing an emergency call versus calling first) may contribute to differential delays, such that EMS activation becomes a key “gatekeeper” step that determines how quickly subsequent resuscitation processes are initiated. Third, from a physiological perspective, bystander CPR can provide partial circulatory support and extend the time window during which definitive care (e.g., defibrillation and advanced life support) remains effective; therefore, delays in EMS activation may become more consequential once CPR is underway [42,43]. Together, these behavioral and physiological considerations suggest that the survival impact of timely EMS activation may be amplified when active bystander intervention is present, consistent with the observed effect modification in our mediation analysis.

Our results align with prior evidence and further support that each minute of delay from collapse to EMS activation subsequently reduces survival and neurological recovery [44,45]. In Sweden, following CPR training in almost 30% of the population between 1990 and 2010, the median time from onset of OHCA to the EMS call dropped from 5 to 2 min, whereas the interval from the arrest to CPR decreased from a median of 10 min to 2 min [46]. Bystanders with CPR training are better than bystanders without training at recognizing the emergent situation of cardiac arrest and taking actions [47]. These national-level initiatives in Sweden, where nearly two million citizens have been trained, have been associated with shorter times from collapse to EMS call and increased bystander CPR, with corresponding improvements in outcomes over time. Together with the current findings, these observations emphasize that both rapid activation and widespread CPR competence are essential to reduce avoidable mortality from OHCA.

Besides a lower bystander CPR rate, those living in socially deprived communities are shown to be less likely to have CPR training [48,49,50]. In the United States, CPR training disparities—such as lower income and lesser educational attainment—are associated with reduced likelihood of CPR training [48]. However, a systematic review suggests that many individuals in socioeconomically deprived areas are willing to learn CPR when training is accessible. Therefore, national and local interventions should support bystander CPR trainings to be more flexible, affordable, and accessible to those in deprived communities [51].

A study by Takei et al. showed delays in emergency calls occurred more frequently in the rural region compared with the central region [28]. Calling others, including family members, relatives, and police, was one of the major reasons for the delay. In another study in Germany, the inability to make a decision to place the emergency call and thinking about what to do were other major reasons suggested that approximately 45% of laypeople are unable to judge if the victim is in cardiac arrest or not [52]. A study in the USA found that in neighborhoods composed of minority and lower SES populations, barriers to calling the emergency services in an OHCA situation include distrust of law enforcement, financial status, language concerns, and lack of recognition of cardiac arrest [53].

Potential strategies to address these disparities include population-level education campaigns focused on recognizing abnormal breathing and cardiac arrest, targeted outreach in deprived communities, optimization of dispatcher protocols for rapid cardiac arrest recognition, and integration of technology-based solutions (such as smartphone emergency apps) to facilitate faster EMS activation. These efforts should particularly target residents of deprived areas, where access to public education and training opportunities remains most limited.

In interpreting these findings, system-level context should be considered when assessing transferability to other countries. Korea operates a nationwide single-access EMS activation system with relatively standardized dispatch processes and linked registry-based data collection, which may influence both baseline performance and variability in early response [17,18]. In other settings, the magnitude of the indirect effect through early EMS activation may differ depending on EMS accessibility and response time, the availability and quality of dispatcher-assisted CPR, population-level CPR training coverage, and social or structural barriers to calling emergency services. These contextual differences may affect the size of observed effects and the feasibility of translating specific interventions. Nevertheless, the conceptual pathway linking early recognition and timely EMS activation to subsequent resuscitation processes represents a foundational mechanism in the Chain of Survival and is likely relevant across diverse emergency care systems.

The modest yet significant mediation proportion observed in this study suggests that delayed recognition of emergencies and calling EMS contribute to, but do not fully account for, the survival disadvantage in deprived areas. Other interacting factors, such as dispatcher response times and resource accessibility, likely compound this effect. Indeed, the mediation effect of ATI was higher among the patients who received bystander CPR supports this concept. These findings highlight complex interactions between social context, lay responder behavior, and emergency system performance, underscoring the critical importance of timeliness in the very first link of the Chain of Survival and that the benefit of rapid activation may be greatest when coupled with active bystander intervention.

This study has several strengths. It is among the first nationwide studies to examine time to EMS call as a mediating factor linking community deprivation and outcomes from OHCA. While previous studies have examined deprivation/disparities in relation to dispatcher recognition time [54], ambulance response time and [37] EMS transport time to ED [55,56], the time to EMS call interval has not been evaluated within a causal framework. Another strength of this study is the use of causal mediation analysis, which allows for the decomposition of the association between regional deprivation and outcomes from OHCA into direct and indirect effects. In addition, the use of a nationally representative registry with over 43,000 OHCA cases enhances the generalization and statistical power of our findings.

Our findings, based on nationwide data from 2015 to 2022, underscore a persistent structural inequity in the prehospital response to OHCA. Although the analyses are limited to data through 2022, these disparities are likely to persist beyond the study period. The recent 2025 ERC Guidelines, which prioritize immediate EMS activation before breathing assessment, are expected to shorten the ATI globally. However, without targeted strategies to mitigate the ongoing ‘digital and health literacy divide’ in socioeconomically deprived areas, improvements in ATI may remain uneven. As more advantaged regions adopt new guidelines more rapidly, disparities in ATI, and therefore in survival outcomes, may paradoxically widen.

Regional deprivation was significantly associated with poorer outcomes, and this association was partially mediated by ATI, even among patients who received bystander CPR. This suggests that although resuscitation knowledge and willingness to perform CPR may exist, delays in recognition and EMS activation persist as critical barriers in deprived communities. Strengthening the initial ‘call-to-action’, in line with the recommendation that “when an adult suddenly collapses, trained or untrained bystanders should, at a minimum, activate their community medical response system” [57], and through community education, accessible emergency communication systems, and equity-focused public health initiatives, is essential to ensure that the benefits of bystander CPR are not offset by delays in EMS activation.

However, this study has several limitations to this study. First, despite the use of a causal mediation approach and adjustment for a comprehensive set of confounders, residual confounding from unmeasured individual or neighborhood-level factors (e.g., health literacy, comorbidity burden, or housing conditions) cannot be excluded. Second, ATI was based on caller-reported times, which may be imprecise in stressful emergency situations; however, consistent patterns across multiple ATI cut-points and sensitivity analyses suggest that any non-differential misclassification is unlikely to fully explain the findings. In addition, dichotomizing ATI at <5 min may have resulted in information loss and may have obscured potential non-linear or threshold effects. We chose this operationalization to maintain clinical interpretability and analytical stability given the skewed distribution and pragmatic constraints of binary mediator specifications in the current mediation framework; importantly, our conclusions remained robust across alternative ATI thresholds (<3, <4, and <10 min). Third, the registry lacked granular information on dispatcher characteristics, public education exposure, or CPR training history (prior knowledge of CPR) of bystanders, limiting deeper exploration of mechanisms underlying social gradients in early response. Finally, the study was conducted within a single national EMS and health system, which may limit generalizability to countries with different emergency care structures or baseline CPR training rates.

## 5. Conclusions

In conclusion, this causal mediation analysis demonstrates that regional deprivation is independently associated with poorer outcomes after OHCA, partly mediated by delays in EMS activation. The mediating effect of delayed EMS activation is more pronounced among patients receiving bystander CPR, underscoring the critical interplay between social context, layperson response and emergency system performance. Strengthening community readiness and bystander engagement remains a crucial strategy to enhance early response and improve survival equity after cardiac arrest.

## Figures and Tables

**Table 1:** Characteristics of the study population by regional deprivation status.

		Total		Non-Deprived Area		Deprived Area	
Total		43,032	100.0	40,390	100.0	2642	100.0
Gender, N (%)							
	Male	31,741	73.8	29,797	73.8	1944	73.6
	Female	11,291	26.2	10,593	26.2	698	26.4
Age Group, mean ± SD				62.5 ± 13.3		65.4 ± 11.9	
Bystander CPR, N (%)							
	Yes	15,268	35.5	14,670	36.3	598	22.6
Shockable Rythm, N (%)							
	Yes	17,719	41.2	16,546	41.0	1173	44.4
ATI < 5 min, N (%)							
	Yes	32,326	75.1	30,535	75.6	1791	67.8
Good Neurological Prognosis, N (%)							
	Yes	5524	12.8	5357	13.3	167	6.3
Survival to Discharge, N (%)							
	Yes	8856	20.6	8485	21.0	371	14.0

Abbreviations: N = number; SD = standard deviation; CPR = cardiopulmonary resuscitation; ATI = awareness time interval.

**Table 2:** Multivariable logistic regression analysis for the clinical outcomes of OHCA, stratified by bystander CPR status.

			Good Neurological Prognosis			Survival to Discharge		
		Total N	Outcome N (%)	Model 1 * aOR (95% CI)	Model 2 ** aOR (95% CI)	Outcome N (%)	Model 1 aOR * (95% CI)	Model 2 aOR ** (95% CI)
Total								
	Reference area	40,390	5357 (13.3%)	Reference	Reference	8485 (21.0%)	Reference	Reference
	Deprived area	2642	167 (6.3%)	0.45 (0.38–0.53)	0.46 (0.39–0.55)	371 (14.0%)	0.63 (0.56–0.71)	0.65 (0.57–0.73)
No Bystander CPR								
	Reference area	25,720	2185 (8.5%)	Reference	Reference	4077 (15.9%)	Reference	Reference
	Deprived area	2044	59 (2.9%)	0.31 (0.23–0.40)	0.31 (0.24–0.41)	204 (10.0%)	0.57 (0.49–0.67)	0.59 (0.50–0.69)
Bystander CPR Performed								
	Reference area	14,670	3172 (21.6%)	Reference	Reference	4408 (30.0%)	Reference	Reference
	Deprived area	598	108 (18.1%)	0.65 (0.51–0.82)	0.67 (0.53–0.85)	167 (27.9%)	0.75 (0.61–0.93)	0.78 (0.64–0.97)

Abbreviations: OHCA = out-of-hospital cardiac arrest; CPR = cardiopulmonary resuscitation; N = number; aOR = adjusted odds ratio; CI = confidence interval. * Model 1: Adjusted for age group, sex, calendar year, insurance type, location of arrest, and initial ECG rhythm. ** Model 2: Additionally adjusted for the awareness time interval as a covariate.

**Table 3:** Causal mediation analysis of the association between regional deprivation and the clinical outcomes of OHCA, stratified by bystander CPR status.

		Total aOR (95% CI)	No Bystander CPR aOR (95% CI)	Bystander CPR aOR (95% CI)
Good neurological prognosis				
	Total Effect	0.44 (0.36–0.52)	0.3 (0.22–0.38)	0.64 (0.49–0.79)
	Natural Direct Effect (NDE)	0.46 (0.38–0.54)	0.31 (0.23–0.4)	0.67 (0.51–0.83)
	Natural Indirect Effect (NIE)	0.96 (0.94–0.97)	0.95 (0.94–0.97)	0.96 (0.93–0.98)
	Proportion Mediated (%, 95% CI)	3.65 (2.09–5.2)	2.09 (0.99–3.19)	7.92 (1.19–14.64)
Survival to discharge				
	Total Effect	0.62 (0.54–0.7)	0.56 (0.47–0.65)	0.75 (0.59–0.9)
	Natural Direct Effect (NDE)	0.65 (0.57–0.73)	0.59 (0.49–0.68)	0.78 (0.62–0.95)
	Natural Indirect Effect (NIE)	0.95 (0.94–0.97)	0.95 (0.94–0.97)	0.95 (0.93–0.98)
	Proportion Mediated (%, 95% CI)	7.88 (4.58–11.18)	6.21 (3.26–9.16)	14.69 (0.73–28.64)

Abbreviations: OHCA = out-of-hospital cardiac arrest; CPR = cardiopulmonary resuscitation; aOR = adjusted odds ratio; CI = confidence interval.

## Data Availability

The data used in this study are provided by the Korea Disease Control and Prevention Agency (KDCA). Any researcher who wishes to access the dataset should obtain permission from the KDCA.

## Supplementary Materials

The following supporting information can be downloaded at: https://www.mdpi.com/article/10.3390/healthcare14030408/s1, Table S1: Characteristics of the study population by regional deprivation status (detailed); Table S2. Statistical association between the independent (regional deprivation) and mediating (achieving <5 min of ATI) variables; Table S3. Sensitivity analyses of the mediating effect of awareness time interval (ATI) on the association between regional deprivation and clinical outcomes after OHCA, stratified by bystander CPR status.

## Abbreviations

The following abbreviations are used in this manuscript:

OHCA	Out-of-hospital cardiac arrest
EMS	Emergency medical services
ILCOR	International Liaison Committee on Resuscitation
ERC	European Resuscitation Council
BLS	Basic Life Support
CPR	Cardiopulmonary resuscitation
KDCA	Korea Disease Control and Prevention Agency
NFA	National Fire Agency
ATI	Awareness time interval
RDI	Regional Deprivation Index
CPC	Cerebral Performance Category
ED	Emergency department
SES	Socioeconomic status
DA	Dispatcher-assisted
