# Supplementary material for: A Moderated Mediation Analysis of Timely EMS Activation and Bystander CPR in the Association Between Regional Deprivation and Outcomes Following Out-of-Hospital Cardiac Arrest
Source: Healthcare (Basel). 2026 Feb 5;14(3):408. doi: 10.3390/healthcare14030408 (PMC12896887; doi:10.3390/healthcare14030408)
Supplement: Supplementary file 1 [file healthcare-14-00408-s001.zip › healthcare-4074256-supplementary.pdf]

Supplementary Table S1. Characteristics of the study population by regional deprivation status (detailed).

	Total		Non-deprived area		Deprived area		p-value
	N	(%)	N	(%)	N	(%)	
Total	43,032	100.0	40,390	100.0	2,642	100.0	
Year of OHCA							<0.05
2015	4,339	10.1	4,142	10.3	197	7.5	
2016	4,810	11.2	4,544	11.3	266	10.1	
2017	5,137	11.9	4,830	12.0	307	11.6	
2018	5,184	12.0	4,842	12.0	342	12.9	
2019	5,189	12.1	4,855	12.0	334	12.6	
2020	5,977	13.9	5,585	13.8	392	14.8	
2021	5,973	13.9	5,597	13.9	376	14.2	
2022	6,423	14.9	5,995	14.8	428	16.2	
Gender, N (%)							0.83
Male	31,741	73.8	29,797	73.8	1,944	73.6	
Female	11,291	26.2	10,593	26.2	698	26.4	
Age Group, N (%)							<0.05
18-30 years	970	2.3	938	2.3	32	1.2	
31-40 years	1,894	4.4	1,844	4.6	50	1.9	
41-50 years	4,870	11.3	4,626	11.5	244	9.2	
51-60 years	9,322	21.7	8,809	21.8	513	19.4	
61-70 years	10,979	25.5	10,302	25.5	677	25.6	
71-80 years	14,997	34.9	13,871	34.3	1,126	42.6	
Insurance Type, N (%)							<0.05
National Health Insurance	37,581	87.3	35,318	87.4	2,263	85.7	
Medical Aid	3,293	7.7	3,087	7.6	206	7.8	
Other/Unknown	2,158	5.0	1,985	4.9	173	6.5	
Location of OHCA, N (%)							<0.05
Public	9,497	22.1	8,989	22.3	508	19.2	
Non-public	22,731	52.8	21,364	52.9	1,367	51.7	
Unknown	10,804	25.1	10,037	24.9	767	29.0	
Bystander CPR, N (%)							<0.05
Yes	15,268	35.5	14,670	36.3	598	22.6	
Shockable Rythm, N (%)							<0.05
Yes	17,719	41.2	16,546	41.0	1,173	44.4	
ATI< 5minutes, N (%)							<0.05
Yes	32,326	75.1	30,535	75.6	1,791	67.8	
Good Neurological Prognosis, N (%)							<0.05
Yes	5,524	12.8	5,357	13.3	167	6.3	
Survival to Discharge, N (%)							<0.05
Yes	8,856	20.6	8,485	21.0	371	14.0	

* Abbreviations: OHCA = out-of-hospital cardiac arrest; N = number; CPR = cardiopulmonary resuscitation; ATI = awareness time interval

Supplementary Table S2. Statistical association between the independent (regional deprivation) and mediating (achieving <5 min of ATI) variables.

	Total, N	Outcome, N (%)	aOR* (95% CI)
Total			
Reference area	40,390	30,535 (75.6%)	Reference
Deprived area	2,642	1,791 (67.8%)	0.67 (0.64-0.76)
No Bystander CPR			
Reference area	25,720	18,765 (73%)	Reference
Deprived area	2,044	1,346 (65.9%)	0.70 (0.64-0.78)
Bystander CPR Performed			
Reference area	14,670	11,770 (80.2%)	Reference
Deprived area	598	445 (74.4%)	0.67 (0.56-0.81)

Abbreviations: ATI = awareness time interval; N = number; aOR = adjusted odds ratio; CI = confidence interval; CPR = cardiopulmonary resuscitation

*Adjusted for age group, sex, calendar year, insurance type, location of arrest, and initial ECG rhythm

Supplementary Table S3. Sensitivity analyses of the mediating effect of awareness time interval (ATI) on the association between regional deprivation and clinical outcomes after OHCA, stratified by bystander CPR status.

	Total aOR (95% CI)	No bystander CPR aOR (95% CI)	Bystander CPR aOR (95% CI)
ATI < 3minutes			
Good neurological prognosis			
Total Effect	0.44 (0.37-0.52)	0.30 (0.22-0.39)	0.64 (0.49-0.79)
Natural Direct Effect (NDE)	0.46 (0.38-0.54)	0.32 (0.23-0.40)	0.67 (0.51-0.82)
Natural Indirect Effect (NIE)	0.96 (0.95-0.97)	0.96 (0.95-0.97)	0.96 (0.94-0.98)
Proportion Mediated (%, 95% CI)	3.37 (1.97-4.76)	1.84 (0.88-2.79)	7.07 (1.07-13.08)
Survival to discharge			
Total Effect	0.63 (0.55-0.70)	0.57 (0.48-0.66)	0.75 (0.59-0.90)
Natural Direct Effect (NDE)	0.65 (0.57-0.73)	0.59 (0.50-0.69)	0.78 (0.61-0.94)
Natural Indirect Effect (NIE)	0.96 (0.95-0.97)	0.96 (0.95-0.97)	0.96 (0.94-0.98)
Proportion Mediated (%, 95% CI)	6.87 (4.00-9.74)	5.34 (2.81-7.88)	11.82 (0.39-23.24)
ATI < 4minutes			
Good neurological prognosis			
Total Effect	0.44 (0.36-0.52)	0.30 (0.22-0.38)	0.64 (0.48-0.79)
Natural Direct Effect (NDE)	0.46 (0.38-0.54)	0.31 (0.23-0.40)	0.66 (0.51-0.82)
Natural Indirect Effect (NIE)	0.95 (0.94-0.97)	0.95 (0.94-0.97)	0.96 (0.93-0.98)
Proportion Mediated (%, 95% CI)	3.73 (2.19-5.28)	2.06 (1.00-3.13)	7.43 (1.00-13.87)
Survival to discharge			
Total Effect	0.62 (0.54-0.7)	0.56 (0.47-0.65)	0.74 (0.59-0.9))
Natural Direct Effect (NDE)	0.65 (0.57-0.73)	0.59 (0.5-0.68)	0.77 (0.61-0.94)
Natural Indirect Effect (NIE)	0.96 (0.95-0.97)	0.96 (0.94-0.97)	0.96 (0.94-0.98)
Proportion Mediated (%, 95% CI)	7.36 (4.29-10.43)	5.88 (3.11-8.66)	11.9 (0.24-23.56)
ATI < 10minutes			
Good neurological prognosis			
Total Effect	0.44 (0.36-0.51)	0.30 (0.22-0.38)	0.61 (0.47-0.76)
Natural Direct Effect (NDE)	0.46 (0.38-0.54)	0.31 (0.22-0.39)	0.67 (0.51-0.83)
Natural Indirect Effect (NIE)	0.96 (0.94-0.97)	0.97 (0.95-0.99)	0.92 (0.87-0.96)
Proportion Mediated (%, 95% CI)	3.59 (1.83-5.34)	1.43 (0.48-2.39)	14.2 (2.98-25.41)
Survival to discharge			
Total Effect	0.61 (0.54-0.69)	0.56 (0.47-0.65)	0.72 (0.56-0.87)
Natural Direct Effect (NDE)	0.64 (0.56-0.72)	0.58 (0.49-0.67)	0.78 (0.62-0.95)
Natural Indirect Effect (NIE)	0.95 (0.94-0.97)	0.96 (0.94-0.98)	0.92 (0.87-0.96)
Proportion Mediated (%, 95% CI)	7.79 (4.24-11.34)	5.01 (2.24-7.79)	23.47 (3.47-43.47)

Abbreviations: aOR = adjusted odds ratio; CI = confidence interval; CPR = cardiopulmonary resuscitation; ATI = awareness time interval
